# Accommodative Lag Persistence in Treated Anisometropic, Strabismic, and Mixed Amblyopia

**DOI:** 10.1155/2022/2133731

**Published:** 2022-05-10

**Authors:** Jit B. Ale Magar, Shaheen P. Shah

**Affiliations:** Ophthalmology Department, Queensland Children's Hospital, Brisbane, Australia

## Abstract

**Background:**

Amblyopic eyes typically exhibit greater lag of accommodation. Whether this improves after amblyopia treatment is inconclusive. The aim of this study is to report post-treatment accommodative response in amblyopia and to investigate if the lag is associated with visual acuity, treatment duration, and amblyopia type.

**Methods:**

Monocular and binocular accommodative responses were measured using Nott's method of dynamic retinoscopy in amblyopia of anisometropic, strabismic, and combined anisometropic-strabismic types and age-matched controls with normal vision. The results were compared using the nonparametric Wilcoxon signed ranks test. Linear regression analysis was used to examine association of the lag to refractive error, duration of therapy, and visual acuity.

**Results:**

Mean ± SD age of 46 amblyopic and 20 control subjects were 6.9 ± 1.8 and 6.9 ± 2.2 years, respectively. At the time of the study, 30 amblyopic subjects were receiving patching therapy and ceased in the remainder. In amblyopic eyes, mean ± SD monocular and binocular lags were 1.2 ± 0.6D and 1.0 ± 0.5D (*p* < 0.001), respectively, compared to 0.6 ± 0.3D and 0.5 ± 0.2D (*p* < 0.005), respectively, in nonamblyopic eyes and 0.4 ± 0.2D and 0.3 ± 0.2D (*p*=0.093), respectively, in the controls. By types, the monocular lag was significantly higher than the binocular lag (*p*=0.001) in mixed amblyopia (*p*=0.004); they were similar in anisometropic (*p*=0.283) and strabismic (*p*=0.743) amblyopia. Monocular lag was significantly correlated to BCVA (*r* = 0.46; *p*=0.001) and refraction (*r* = 0.42; *p*=0.001) but not to patching duration (*r* = 0.1; *p*=0.280).

**Conclusion:**

Inadequate accommodative response, a higher lag, persists in amblyopic eyes even after the treatment. Impaired accommodative response is partly determined by posttherapy visual acuity. Further studies investigating the effect of accommodative lag on visual recovery and whether optical correction of the deficiency may improve visual outcome of the treatment are recommended.

## 1. Background

Amblyopia is a visual impairment in an otherwise structurally normal eye [1] affecting 2–5% of the population [2, 3]. The condition is one of the most common reasons for referral to a paediatric ophthalmology service [4]. In addition to reduced visual acuity, amblyopia causes deprived higher-order visual functions [5] with adverse impact in their quality of life [6, 7].

A minimum level of accommodative accuracy to a demand is required in order to place a clear image of a near object of regard on the retina. When an eye underaccommodates, referred as accommodative lag, or overaccommodates, referred as the accommodative lead, outside the limit of physiological normal depth-of-focus (typically ± 0.5D [8]), the near blur caused by hyperopic defocus is visually noticeable [9]. This blur may lead to regression of visual acuity [10, 11].

A higher lag of accommodation in amblyopic eye has been consistently reported in the literature [12–18]. The lag has been reported to be higher in monocular than binocular viewing conditions [17]. Although an exact aetiology is unknown, the anomaly is likely associated with an early and prolonged abnormal visual experience in the sensory visual system [19].

Whether accommodative function improves with treatment of amblyopia is uncertain. While some reported improved accommodative function following treatment [20], others found it to be individually inconsistent [17]. The reason for inconsistency is not fully known. The aim of this study was to measure the static accommodative response in children currently undergoing or have undergone amblyopia treatment and to evaluate whether this is associated with refractive status, duration of treatment, posttreatment visual acuity, and types of amblyopia. The results from the amblyopic eye, the fellow nonamblyopic eye, and the eyes in age-matched children with normal vision were compared.

## 2. Methods

This single centre study was approved by institutional ethics review board (reference: HREC/21/QCHQ/74840) and followed ethical principles according to the tenets of the Declaration of Helsinki. Informed consent from parents or guardians of all subjects was obtained before enrolment.

Patients diagnosed with amblyopia aged between 4 and 10 years presented in the paediatric ophthalmology clinic (between May 2020 and March 2021) were consecutively enrolled after obtaining informed consent. Amblyopia was defined as the difference in best-corrected visual acuity between the eyes by at least two Snellen's acuity levels without intrinsic pathology on examination. The amblyopia consisted one of anisometropic, strabismic, or mixed (with strabismus and anisometropia) types. Children with sensory deprivation amblyopia were excluded. At the time of the study visit, all amblyopic subjects were either actively receiving or had previously received occlusion therapy. The therapy comprised of patching and/or atropine penalisation along with refractive correction as required. Individuals currently receiving atropine or those who had cycloplegic agents within one month from the study visit were excluded. Myopia exceeding −5.0D, inability to perform the study-related clinical tests, neurological disorders, Down's syndrome, developmental delay, previous ocular surgery, and general health conditions that might affect vision or muscular functions were also excluded. The control group consisted of patients visiting the clinic for follow-up of resolved anterior surface or adnexal disorders such as allergic conjunctivitis, chalazion, lacrimal system disorders, and blepharitis. Subjects in this group had the best-corrected visual acuity (BCVA) of 0.1 logMAR or better in each eye with refraction not exceeding ±2.0D spherical, 1.50D astigmatism, and 1.0D anisometropia. Subjects wore full refractive correction at the time of the study examination. Both groups had otherwise normal eye examination.

Visual acuity was assessed using logMAR single optotypes, letters, or Lea symbols, with crowding bars projected on a digital screen (Medmont Mate AT20R, Medmont International) at a 3-meter distance. Nott's method of near retinoscopy [21] was used to determine binocular and monocular accommodative responses by a single examiner (JM). The technique is highly reproducible and repeatable [22] and produces similar result as automated [23] and other methods of dynamic retinoscopy [24]. In this procedure, with distance correction on, if any, the subjects viewed to a high contrast target (6/9 size dark black letters on white background) at 25 cm (dioptric value of 4.0D) fixation distance which represents habitual working distance for children [25]. Subjects were required to read the letters with both eyes open during the assessment of binocular lag. For the assessment of monocular lag, the nonexamining eye was occluded using a sticky patch over the glasses, if wearing, or a pirate patch, if not wearing spectacles.

To ensure “on-axis” measurement of the lag, retinoscopy and visual axes were maintained as closely parallel as possible during the retinoscopy. Examiner evaluated the retinoscopy reflex along the vertical meridian from the fixation target using a streak retinoscope (WelchAllyn, Skaneateles Falls, NY, USA). Depending on “with” or “against” motion of the retinoscopy reflex, the working distance was increased by moving away or decreased by approaching closer to the patient's eye until neutralisation of the reflex was achieved. The RAF rule was used to maintain and measure the distances. Dioptric value of the distance of neutrality was recorded as accommodative response. The difference between the accommodative response and the demand was defined as the lag (response < demand) or the lead (response > demand) of accommodation.

Descriptive data including age, BCVA, refraction, and lag were reported using mean, standard deviation, and range or interquartile (25^th^ and 75^th^ percentile) as appropriate. The nonparametric Wilcoxon signed ranks test was used to compare the variables between the amblyopic and the fellow eyes and between the groups. The linear regression analysis was used to examine association of accommodative response with amblyopia type, refractive error, age, duration of occlusion therapy, and visual acuity.

## 3. Results

Demographics and clinical characteristics of the total 46 amblyopic and 20 control subjects are given in Table 1. The mean ± SD age of amblyopic and control subjects were 6.9 ± 1.8 and 6.9 ± 2.2 years, respectively. Of the amblyopic subjects, 30.4% (*n* = 14) were anisometropic (difference in refraction by ≥ 1.25D between the eyes), 43.5% (*n* = 20) were mixed, and 26.10% (*n* = 12) were strabismic types. Spherical equivalent refraction (SER) in the amblyopic eyes was significantly different from the nonamblyopic eyes in anisometropic (+5.6 vs. + 2.6D; *p* < 0.001) and mixed (+6.1 vs + 3.2D; *p* < 0.027) types, but similar (+1.7 vs. + 1.3D; *p*=0.121) in strabismic amblyopia. All eyes in anisometropic and mixed types of amblyopia had hyperopic SER. The control children had no interocular difference in SER (*p*=0.893); mean ± SD SER were 0.5 ± 0.6D in the right and 0.6 ± 0.6D in the left eye.

Thirty amblyopic subjects were actively receiving patching therapy with the mean ± SD duration of 11.8 ± 6.6 months (median = 9; range 3–26 months). Occlusion had been ceased in the remaining 16 subjects after the mean ± SD patching duration of 17.6 ± 5.0 months (range 10 to 25 months). Four subjects had atropine penalisation at some stage for the duration of 4–7 months due to noncompliance to patching.

The mean ± SD BCVA in the amblyopic eyes (0.50 ± 0.24 logMAR) was significantly different (*p* < 0.001) to the fellow eye vision (0.10 ± 0.10 logMAR). The BCVA in anisometropic, mixed, and strabismic subjects was 0.53 ± 0.28, 0.47 ± 0.20, and 0.53 ± 0.25 log MAR, respectively, in the amblyopic eye and 0.07 ± 0.08, 0.12 ± 0.11, and 09 ± 0.09 logMAR, respectively, in the fellow eye. Except for one anisometropic subject who had visual acuity of 0.10 logMAR in the amblyopic eye (0.00 logMAR in the fellow eye), the BCVA in all other amblyopic eyes was ≤0.20 logMAR with at least two Snellen's acuity level worse than in the fellow eyes. The control group children had equal right and left mean ± SD BCVA of −0.02 ± 0.04 logMAR. There was no statistical difference in amblyopic eye BCVA by types of amblyopia (*p*=0.673).

**Figure 1:**
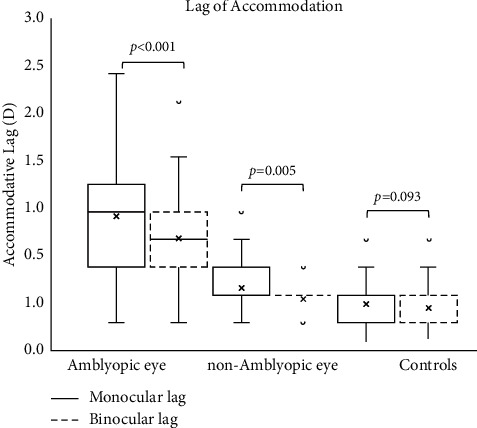
Binocular and monocular accommodative lags in amblyopic, fellow, and control eyes. Horizontal line and marker within the boxes represent median and mean, respectively.

The mean ± SD binocular accommodative lag in the amblyopic eye, fellow eye, and controls was 1.0 ± 0.5D, 0.5 ± 0.2D, and 0.3 ± 0.2D, respectively. Similarly, the mean ± SD monocular accommodative lag in amblyopic, nonamblyopic, and controls eyes was 1.2 ± 0.6D, 0.6 ± 0.3D, and 0.4 ± 0.2D, respectively (Figure 1). Both binocular and monocular lag in the amblyopic eye was significantly higher than in the fellow eye and in controls (*p* < 0.001). The difference in monocular lag between the nonamblyopic eye of amblyopic subjects and controls was also statistically significant (*p*=0.023).

Only mixed amblyopia showed significantly greater monocular than binocular lag (*p*=0.004); there were no differences in anisometropic (*p*=0.283) and strabismic (*p*=0.743) amblyopia. Accommodative lead was not found in any child included in this study. Results of the monocular and binocular lag of accommodation by amblyopia types are given in Table 2.

**Figure 2:**
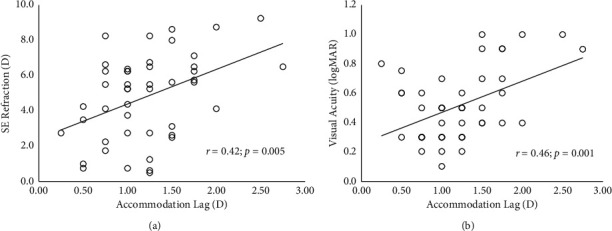
Correlation of monocular accommodation lag with spherical equivalent (SE) refraction (a) and visual acuity (b) of the amblyopic eye. Some data points (circles) may represent over-laid multiple samples.

On regression analysis, both binocular and monocular accommodative lags in amblyopic eye were moderately correlated to BCVA and SER.  Figure 2 shows the correlation of monocular accommodation lag with BCVA (*r* = 0.46; *p*=0.001) and refraction (*r* = 0.42; *p*=0.005). Correlation of the lag to the age at diagnosis (*r* = 0.1; *p*=0.229), age at the time of examination (*r* = 0.1; *p*=0.226), and patching duration (*r* = 0.1; *p*=0.280) were not significant.

## 4. Discussion

Any factor that might degrade retinal image quality during the critical period of vision development results in an insufficient sensory stimulation to the visual cortex resulting in amblyopia [26]. In some cases, abnormal accommodation alone may be amblyogenic [20]. Therefore, the primary requirement in amblyopia treatment is to ensure optimum visual stimulation by eliminating any optical defocus. Our study found binocular accommodative lag in excess of 1.0D in majority (63%) of the treated amblyopic eyes which may be optically significant (≥1.0D defocus is regarded as greater than the resolution limit [27, 28]) as the amount exceeds the critical limit of the foveal depth-of-focus [8]. Consistent with a previous report [17], we also found reduced accommodative response (greater lag) in the fellow nonamblyopic eye compared to that in unaffected children (0.6D vs. 0.4D, *p*=0.023).

Over 80% of the visual improvement in amblyopia occurs within first six weeks (over 90% in 12 weeks) of patching [29]. Our amblyopic subjects had received the treatment for the mean duration of over a year, and we can therefore suggest that they were fully treated. Among our amblyopic subjects, while the level of visual acuity significantly predicted accommodative lag, it was essentially independent of the duration of the treatment. Thus, an optimum effect of the therapy may have already been established. This could be the reason for insignificant correlation between the patching duration and the accommodative lag. Nevertheless, the significant correlation between visual acuity and accommodative lag in amblyopic eye supports the sensory hypothesis, rather than motor hypothesis, and that accommodative response is more accurate with improvement in sensory input (i.e., better accommodative response in eyes with better vision).

Our results are consistent with other studies reporting that the monocular accommodative lag was greater than the binocular lag [17]. This appeared more exaggerated in the mixed type of amblyopia. The consensual accommodation between the eyes [30] may be responsible for a more accurate binocular accommodative performance, in which equal amount of response may have been driven by the accommodative effort in the nonamblyopic eye as proposed previously [17]. However, the reason for similar (statistically indifferent) monocular and binocular lags in anisometropic and strabismic amblyopia, but not in mixed type, is not clear. Further study with larger sample size may elicit a more definitive difference.

Ciuffreda et al. demonstrated reduced slope of the stimulus/response accommodative performance curve in amblyopia potentially attributed to increased depth-of-focus [31]. Accommodative response is closely associated with the depth-of-focus, which in turn is determined by number of optical, anatomical, and physiological factors including visual acuity, refractive error, pupil size, contrast, and retinal eccentricity [32]. Assuming the pupil size constant and since we assessed on-axis accommodative response with full refractive correction under a controlled room and target illumination, the visual acuity remains only relevant factor affecting depth-of-focus in this study. Among the amblyopic subjects, vision in the affected eye was worse by at least 1 Snellen's acuity level compared to that in the fellow eye, and essentially all amblyopic eyes had visual acuity ≤ 0.20 logMAR (only one subject had better vision). An eye with a poor vision may not require to fully accommodate to the theoretical demand (determined by target distance) as needed to resolve a finer detail (high resolution target). We used high resolution target requiring to resolve them (be able to read) which ensured maximum accommodation being used during the assessment. Therefore, although our results of underaccommodation in the amblyopic eyes may be attributable to increased depth-of-focus related to reduced visual acuity to some extent, this does not necessarily eliminate the possibility of defective accommodative response as a primary deficit.

A limitation of this study is that we did not measure accommodative lag prior to initiation of the amblyopia treatment. A temporal relationship (prior to, during and posttreatment) between the accommodative response and visual acuity would provide a better understanding of the underlying mechanisms. Furthermore, although Nott's retinoscopy is highly reliable, the accuracy may be compromised particularly during the assessment of binocular lag. When assessing the response in the amblyopic eye, the consensual accommodation driven by the fellow nonamblyopic eye (fixating eye) may result in overestimation of the response. Similarly, while assessing the response in the fellow eye, the retinoscope light may disrupt accommodation resulting in underestimation. Therefore, an automated dynamic refractor would have been ideal for this purpose. The accommodative response also depends on binocular vision status [33]. Analysis of potential relationship between stereoacuity and accommodative response would have been beneficial.

In conclusion, our study found persisting lag of accommodation in treated amblyopia. The amount of lag was predicted by the level of posttreatment visual acuity and refractive error. Further studies are recommended to investigate whether optical correction of the lag, such as using bifocals, to reduce the lag induced blur within the limit of the physiological depth-of-focus (foveal limit of resolution) may improve amblyopia treatment outcomes.

## Figures and Tables

**Table 1:** Subject demographics and clinical characteristics (mean ± SD; range).

	Anisometropic	Mixed	Strabismic	Controls
*n*	14	20	12	20
Age (y)	7.2 ± 2.0; 4–10	7.1 ± 1.7; 5–10	6.3 ± 1.8; 4–10	6.9 ± 2.2; 4–10
Diagnosis age	5.0 ± 2.6; 1–9	4.6 ± 2.0; 2–9	3.3 ± 2.4; 1–9	NA
Patched (month)	10.1 ± 4.6; 3–20	14.1 ± 6.6; 3–25	17.6 ± 6.9; 5–26	NA
Refraction (SE)				
Amblyopic	5.6 ± 1.5; 2.8–9.3	6.1 ± 1.8; 2.6–8.8	1.4 ± 1.1; 0.5–3.5	NA
Nonamblyopic	2.6 ± 1.7; 0.3–5.3	5.7 ± 1.9; 1.6–8.3	1.3 ± 0.9; 0.3–2.5	0.5 ± 0.6; −0.5–+1.5^*∗*^
BCVA				
Amblyopic	0.5 ± 0.3; 0.1–1.0	0.5 ± 0.2; 0.2–1.0	0.5 ± 0.3; 0.2–1.0	NA
Nonamblyopic	0.1 ± 0.1; 0.0–0.2	0.1 ± 0.1; 0.0–0.3	0.1 ± 0.1; 0.0–0.3	0.0 ± 0.04; −0.1–0.0^*∗*^

SE, spherical equivalent; BCVA, best-corrected logMAR visual acuity. ^*∗*^Right eye data.

**Table 2:** Monocular and binocular lag of accommodation by type of amblyopia.

	Lag (D)	Amblyopic, mean ± SD (IQR)	Nonamblyopic, mean ± SD (IQR)	*P* value (two-tailed)
Anisometropic	Monocular Binocular	1.2 ± 0.8 (0.9, 1.8) 1.2 ± 0.5, (0.8, 1.6)	0.7 ± 0.1, (0.5, 0.8) 0.4 ± 0.1, (0.3, 0.5)	0.012 0.001

Mixed	Monocular Binocular	1.3 ± 0.5, (1.0, 1.5) 1.0 ± 0.3, (1.0, 1.3)	0.6 ± 0.3, (0.5, 0.8) 0.5 ± 0.2, (0.3, 0.5)	<0.001 <0.001

Strabismic	Monocular Binocular	0.9 ± 0.4, (0.5, 1.3) 0.8 ± 0.8, (0.3, 1.5)	0.7 ± 0.3, (0.5, 0.9) 0.6 ± 0.2, (0.5, 0.8)	0.142 0.151

*P* values represent significance of the difference between amblyopic and nonamblyopic eyes.

## Data Availability

The data used to support the findings of this study are available from the corresponding author upon request.
